# A Case of Enteric Fever, with Remarks

**Published:** 1861-02

**Authors:** G. V. Ewing

**Affiliations:** Rock Run, Ill.


### A CASE OF ENTERIC FEVER, WITH REMARKS.

BY G. V. EWING, M. D., ROCK RUN, ILL.

In the following case, we do not claim that there is anything peculiar in the symptoms, aside from what is usually seen in "Enteric Fever." Neither is it claimed, that there is anything new in the use of the remedy to which we wish to direct particular attention in the treatment of this form of fever, but rather to re-establish the use of an old remedy which has, to a very great extent, fallen into unmerited neglect.

December 7th, 1859, I was requested to visit C. K——p, a German boy, aged twelve years, who, the messenger informed me, had been indisposed for two weeks, the symptoms being premonitory of fever, languor, copos, formication, pain in extremities, loss of appetite, headache, chills, fever and diarrhœa. All these symptoms had been increasing in violence from the first onset up to the period when I was called, when I found the patient in the following condition: high fever, pulse running up to 130 in the minute, tongue coated, sordes on lips and teeth, frequent watery discharges from the bowels, abdomen tympanitic, great tenderness in right iliac fossa, urine scanty and highly colored, skin hot and dry, subsultus tendinum, delirium; has not slept any for forty-eight hours.

℞. Pul. Doveri, gr. 12;
Tannin, " 10;
" Opii, " 3;
Hyd. C. Cret. " 10;
M.—In chartulas sex. divid.

One to be taken every four hours in syrup. A teaspoonful of Turpentine Emulsion, with twenty drops Tinct. Valerian, halfway between the powders to be taken. Also, ordered cold to the head and warm fomentations to the bowels.

Dec. 8th—The patient slept some during last night; the symptoms continue about the same. Treatment continued.

Dec. 10th—Not so much diarrhœa as at last date; the other symptoms continue the same. With the addition of a gr. Pul. Camphor to each powder, the treatment was continued.

Dec. 11th—The patient has some refreshing sleep; no material change in the symptoms. Treatment continued.

Dec. 12th—Since yesterday, the patient has had involuntary discharges from the bowels and bladder. Prescribed two grains of Camphor with each powder, to be continued with the other treatment as before.

Dec. 14th—This morning the patient is slightly ptyalized. The symptoms all more favorable; bowels less tympanitic; discharges voluntary and not so frequent; sordes cleaning off the lips and teeth; no delirium; subsultus gone; pulse down to 90. From this date, the patient slowly, but gradually, improved, and on the 21st was discharged from my visiting list, being about twenty-eight days from the first manifestation of the symptoms.

As it is my purpose to direct attention specially to but one feature of the case, (the operation of mercury,) I have condensed the report as much as possible. And I may be permitted to remark here, that I attribute a most decided effect to the operation of the mercury in this case. By a reference to the case, we find no evidence of permanent improvement, until on the eighth day from the commencement of the treatment, when the constitutional effects of mercury were manifested, and that from that date, the improvement in the symptoms was gradual and permanent. But it must not be supposed that this is an isolated case, in which the good effects of mercury were manifest, and that from it alone our conclusions have been drawn. We do not claim the right to draw conclusions from such limited experience, and establish them as principles of practice; but from an extensive experience in the treatment of "Enteric Fever," I have settled down upon this as a rule, with but few exceptions, that as soon as my patient gives evidence of coming under the constitutional effects of mercury, do I feel warranted in pronouncing a favorable prognosis.

During the past few years, this disease has prevailed very extensively in the Northwest, and in some communities it has been very fatal. In some cases, almost entire communities have been prostrated. So common, indeed, is the disease over the western prairies, that it may properly be considered the popular disease of the times, notwithstanding the dishonorable use which irregular physicians are making of the term Typhoid Fever.

In one instance, I attended eight cases in one family, and on the opposite side of the street, four cases in another family, and within two miles of this point, some fifteen others, all of them being well marked, and unmistakable cases of Enteric Fever.

In all these, a very similar course of treatment was adopted, as in the case reported above. In every case, during the past season, we employed the mercurial treatment, and in only one case was the termination fatal, and in only four did the disease run over twenty-one days after the stage of incubation. In these the effects of the mercury could not be secured.

We are sensible of the fact, that at the present time many of our regular and respectable physicians are discarding this medicine from their list of remedial agents. Whilst we cannot look with any degree of complacency upon "old fogyism," we are also free to confess, that we are not fully adequate to the task of keeping pace with the rapid strides of "Young America." The time was when the *lancet*, *calomel* and the *purge* were the "ne plus ultra" of all medical practice. But in honor to the investigating spirit of the profession, for the last score and half years, this practice has been greatly modified. Every physician of observation knows, and will admit, that mercury, especially in fevers, has been greatly abused. For the injudicious use of this, as well as any other article of the materia medica, the physician, and not the remedy, is to blame. In the hands of some doctors, mercury has no doubt "done harm."

There are, perhaps, but very few of the members of the profession at this day, who will contend that excessive salivation is ever requisite.

There is a point beyond which the remedy should never be carried, and that point has been very properly designated by the term "sub-ptyalism," and is indicated by its first impression upon the gums. At this point the medicine should be immediately suspended, or if it is desired to keep up the impression for a few day, it should be done with great caution.

In cases in which it is necessary to evacuate the bowels at the outset of the treatment, the safest and most efficient for that purpose, is calomel and oil. In the disease under consideration, the indications for the use of mercury are not based upon mere hypothesis. When the vital action has abated, which may be very soon after the patient is brought down, usually some degree of delirium or stupor supervenes; the tongue and skin become dry; the bowels become tympanitic; the urine scanty and high colored; the pulse more frequent and feeble. At this juncture, there is great danger of disorganization of certain organs. Every physician knows how very unmanageable the disease sometimes becomes at this stage. Under these circumstances, there is perhaps no remedy so efficient as mercury given to the extent referred to. Its antiphlogistic properties—the general failure of the secretions, the languid condition of the vital functions, all indicate the remedy.

Its effects upon the glands of Peyer, in arresting the progress of the disease and hastening resolution, cannot be doubted. As an objection to the use of mercury in these cases, it is urged, that it acts as a local irritant to those already irritated and diseased patches; but we look upon this as a mistaken idea. It cannot act as an irritant if it is properly guarded by astringents and opiates, which are most generally indicated and constitute a very important part of the treatment.

In regard to the claims of mercury in the treatment of Enteric Fevers, we do not wish to lose sight of other remedies; opiates, tonics, the vegetable and mineral astringents, stimulants, and turpentine, all hold important places in the treatment.

If experience and observation are worth anything at all, we are constrained to claim for mercury in this disease all that has been urged in its favor by many of our most eminent American and European authors and practitioners. In the treatment of this disease, there is great discrepancy of opinion. Perhaps if there was greater uniformity in the treatment, as founded upon rational views of its nature, the disease would be divested of much of its terror.

In visiting the sick, we almost dread to pronounce our diagnosis of a case of Typhoid Fever. The prospect of four, six, or even ten weeks illness, excites a sigh that is painful to the sensitive ear. Every facility for arresting the progress of such a disease, is worthy of at least a passing notice. "But when doctors differ, who is to decide?"

